# Impact of Circulating Estrogen and Mild Systemic Dehydration on Ultrasonic Vocalizations in Female Rats

**DOI:** 10.1044/2026_JSLHR-25-00912

**Published:** 2026-05-26

**Authors:** Kaitlyn Dwenger, Naila Cannes do Nascimento, Taylor W. Bailey, Jun Xie, Abigail Cox, Allison J. Schaser, M. Preeti Sivasankar

**Affiliations:** a Department of Speech, Language, and Hearing Sciences, Purdue University, West Lafayette, IN; b Department of Statistics, Purdue University, West Lafayette, IN; c Department of Comparative Pathobiology, Purdue University, West Lafayette, IN

## Abstract

**Purpose:**

Hormonal and hydration states are known to individually influence laryngeal physiology, yet their distinct and combined effects on vocal function remain poorly understood. This study investigated the interaction of reduced circulating estrogen, estradiol replacement, and mild systemic dehydration (DEHY) on ultrasonic vocalization (USV) in adult female rats.

**Method:**

Forty-eight Long–Evans rats were assigned to six groups (*n* = 8 per group) that differed in hormonal state (control, ovariectomized + estradiol pellet [OVX + Estradiol], OVX + placebo pellet) and hydration status (euhydration [EUHY], DEHY). USVs were recorded during a food-reward elicitation paradigm before hormonal (pellet) replacement, after hormonal replacement, and after 5 days of EUHY or DEHY. Dependent measures included USV number, duration, frequency range, and maximum power. Physiological measures (body weight, water intake, kidney renin expression, uterine weight) supported experimental manipulations.

**Results:**

OVX animals produced shorter duration and lower power USVs compared with controls, although total number and frequency range were largely preserved. Even after hormonal replacement, OVX + Estradiol animals emitted fewer USVs with shorter duration, narrower frequency range, and reduced power as compared with control animals. DEHY narrowed USV frequency range across hormonal groups and interacted with hormonal status to modulate USV number and duration.

**Conclusions:**

Both circulating estrogen and systemic hydration status selectively influenced USV profiles. Although statistically significant, USV changes were generally of small magnitude, warranting caution in interpretation. These findings highlight the nuanced and context-dependent nature of endocrine and hydration influences on vocalizations and establish a preclinical framework for investigating hormone–hydration interactions in laryngeal function.

Hormonal fluctuations of estrogen and other sex hormones exert a significant influence on voice quality throughout the lifespan, notably during menopause or in instances of hormone replacement (Bhatia et al., 2025; Lã & Ardura, 2022; Zamponi et al., 2021). Estrogen also plays a vital role in regulating body hydration and affecting various physiological processes, including fluid balance, renal function, cardiovascular regulation, and thermoregulation (Barron et al., 1986; Curtis, 2009; Santollo & Daniels, 2015; Sladek & Somponpun, 2008; Stachenfeld, 2008; Stachenfeld et al., 2001). Despite these documented systemic effects, the precise role of estrogen in impacting laryngeal physiology remains inadequately understood. Estrogen has been implicated in maintaining vocal fold health, indicating that depletion in circulating estrogen could impact vocalization (Baltgalvis et al., 2010; Kim, Kim, et al., 2020; Kim, Shin, et al., 2020). Estradiol deficiency has been associated with changes in laryngeal tissue viscoelasticity, including decreased expression of hyaluronic acid, collagen, and elastin, alterations that may also compromise laryngeal physiology (Abitbol et al., 1999; Kim, Shin, et al., 2020; Surmeli et al., 2011).

Changes to vocal fold hydration state also affect laryngeal physiology. Systemic hydration is essential for maintaining vocal health (Alves et al., 2019; Hartley & Thibeault, 2014; Sivasankar & Leydon, 2010). In addition, systemic dehydration (DEHY) leads to molecular and structural alterations in the vocal folds, including the disruption of epithelial integrity, changes in extracellular matrix composition, and the upregulation of inflammatory markers (Bailey, do Nascimento, dos Santos, Sivasankar, & Cox, 2023; Cox et al., 2021; do Nascimento, Bailey, et al., 2022; do Nascimento et al., 2020; Duan et al., 2021; Venkatraman et al., 2022). These alterations can compromise the viscoelastic properties of vocal fold tissue, which are essential for healthy phonation (Duan et al., 2021; Miri, 2014). Systemic rehydration has been demonstrated to reverse some of these effects by restoring tissue properties and voice quality in both animal and human studies (Alves et al., 2019; Bailey, do Nascimento, dos Santos, Cox, & Sivasankar, 2023; Bailey, do Nascimento, dos Santos, Sivasankar, & Cox, 2023; Oleson et al., 2020; Pearl Solomon & Stemmle DiMattia, 2000). Even mild DEHY can alter gene expression profiles and laryngeal biology, although these changes may not always translate to detectable differences in vocalization, highlighting the sensitivity of the larynx and vocal function to hydration status (Duan et al., 2021; Oleson et al., 2020; Rodgers et al., 2025). Despite substantial evidence linking sex hormones to fluid regulation and mucosal homeostasis (Barron et al., 1986; Stachenfeld, 2008; Stachenfeld et al., 2001), the interaction between estrogen and DEHY in the context of vocal physiology remains insufficiently explored. Estrogen's role in fluid balance and mucosal hydration may influence laryngeal tissue compliance and neuromuscular excitability, thereby shaping both the likelihood of initiating vocalization and acoustic structure. DEHY could exacerbate the effects of estrogen depletion by further reducing tissue viscous properties and disrupting tissue elasticity.

The intersection of estrogen regulation and systemic DEHY-induced biological changes presents a compelling framework for investigating their joint influence on laryngeal physiology. Collectively, preclinical studies suggest that estrogen helps maintain laryngeal health and tissue viscoelasticity, while DEHY impacts epithelial repair and inflammatory responses, but the underlying combined mechanisms remain unclear, especially as they relate to and impact laryngeal physiology and vocalizations. Carefully controlled studies that manipulate both estrogen and hydration levels are needed to elucidate how these systems interact to shape laryngeal function (i.e., vocalizations). However, there is difficulty conducting carefully controlled, systematic investigations of the influences of estrogen and DEHY on vocalizations using human subjects. Thus, animal models offer a powerful approach for examining these variables that are difficult to isolate in human studies.

Rats provide an ideal animal model for studying how hormonal manipulations and hydration perturbations, via systemic challenges, interact to shape vocal behavior (Cox et al., 2021; Duan et al., 2021; Kim, Shin, et al., 2020; Lenell & Johnson, 2021; Oleson et al., 2018). The well-characterized anatomy and neuromuscular control of the rat larynx support detailed, in vivo analyses of laryngeal function under experimentally controlled conditions (Johnson et al., 2010; Park et al., 2024; Riede, 2011; Riede et al., 2017). While the rat estrous cycle does not directly parallel the human menstrual cycle, it exhibits conserved features of cyclical ovarian hormonal regulation. Both species show phase-dependent fluctuations in estradiol and progesterone, although the timing and duration of progesterone elevation differ substantially (e.g., periovulatory in rats vs. luteal phase in humans; Chaffin & VandeVoort, 2013; Lu et al., 1979; Sato et al., 2016). These features make rats a useful, but not completely homologous, model for investigating estrogen effects. Previous studies have leveraged rodents, including rats, to explore hormone-driven changes in vocal fold biology and vocal behavior (Baltgalvis et al., 2010; Kim, Shin, et al., 2020) as well as systemic DEHY and its impact on laryngeal biology (Cox et al., 2021; Duan et al., 2021; Rodgers et al., 2025).

Ultrasonic vocalizations (USVs), which reflect both physiological and behavioral states, provide a sensitive and quantifiable outcome of laryngeal function (Brudzynski, 2005; Johnson et al., 2010; Kelm-Nelson et al., 2018; Lenell et al., 2021; Shembel et al., 2022). Fifty-kilohertz USVs, typically associated with prosocial or appetitive contexts, require precise neuromuscular coordination of the intrinsic laryngeal muscles and are generated through whistle-like mechanisms involving glottal constriction and high-frequency airflow control (Johnson et al., 2010; McGinnis & Vakulenko, 2003; Riede, 2011; Riede et al., 2017; Wöhr et al., 2008). As such, these vocalizations are highly sensitive to structural and functional changes in the larynx, possibly including those driven by hormonal or hydration-related shifts. However, unlike humans, rat vocal folds do not undergo true vibratory phonation during USV production. Consequently, findings in USV outcomes primarily reflect neuromuscular coordination and laryngeal function, rather than direct biomechanical properties. While prior work has largely examined hormonal effects on USVs in reproductive contexts, showing that estrous status, ovariectomy, and hormone replacement strongly influence USV number and acoustic features (Garcia et al., 2017; Lenell & Johnson, 2021; Matochik et al., 1992; Thomas & Barfield, 1985; Wöhr & Schwarting, 2013), these studies have focused almost exclusively on mating-related behaviors. In contrast, evidence outside reproductive contexts is limited, with estradiol replacement producing mixed or even suppressive effects on USV number, depending on the social context (Garcia et al., 2017; Lenell & Johnson, 2021). Notably, Lenell and Johnson investigated USVs outside mating contexts in social isolation and dyad conditions and found that estrous cycle stage influences USV acoustic parameters, even in these nonreproductive scenarios. However, hydration is rarely considered, despite its known impact on laryngeal physiology. Mild DEHY alone does not appear to alter USVs collected in a nonreproductive context (Rodgers et al., 2025). Whether combined hormonal and hydration states influence vocal behavior remains unknown. Thus, unlike prior work centered on mating paradigms or reproductive behaviors, the present study specifically investigates the interaction of estrogen and hydration states on USVs in nonsocial, nonreproductive contexts.

While prior work has examined estrogen manipulation or hydration independently, this study was designed a priori to replicate established estrogen effects within a food-reward (nonsocial, nonreproductive) USV paradigm and extend this work by directly testing interactions between circulating estrogen and hydration on laryngeal function. The overarching goal of this study was to understand the interaction of circulating estrogen and mild systemic DEHY on the USV profile of 48 adult female rats. USVs were assessed using a food-reward elicitation paradigm in female rats that underwent ovariectomy (surgical removal of the ovaries to eliminate endogenous ovarian hormone production) before and after estradiol (ovariectomized [OVX] + Estradiol) or placebo (OVX + Placebo) replacement. Estradiol, the primary biologically active form of estrogen in cycling females, was administered to model circulating estrogen replacement in the absence of ovarian cycling. Female rats with intact ovaries served as controls. The food-reward USV elicitation method reduced motivation confounds associated with social and mating conditions, where estrogen can influence drive, receptivity, and arousal, complicating the interpretation of USV outcomes. The OVX + Estradiol group isolates the effects of exogenous estradiol without ovarian cycling, allowing comparison with estrogen-depleted (OVX) and hormonally intact (control) animals. This design distinguishes baseline estradiol effects from natural cyclical fluctuations, and OVX + Estradiol animals may still differ from controls due to the absence of other ovarian hormones and typical hormone cycles. All three groups of female rats (controls, OVX + Estradiol, OVX + Placebo) then underwent DEHY or euhydration (EUHY; see Figure 1).

**Figure 1.**
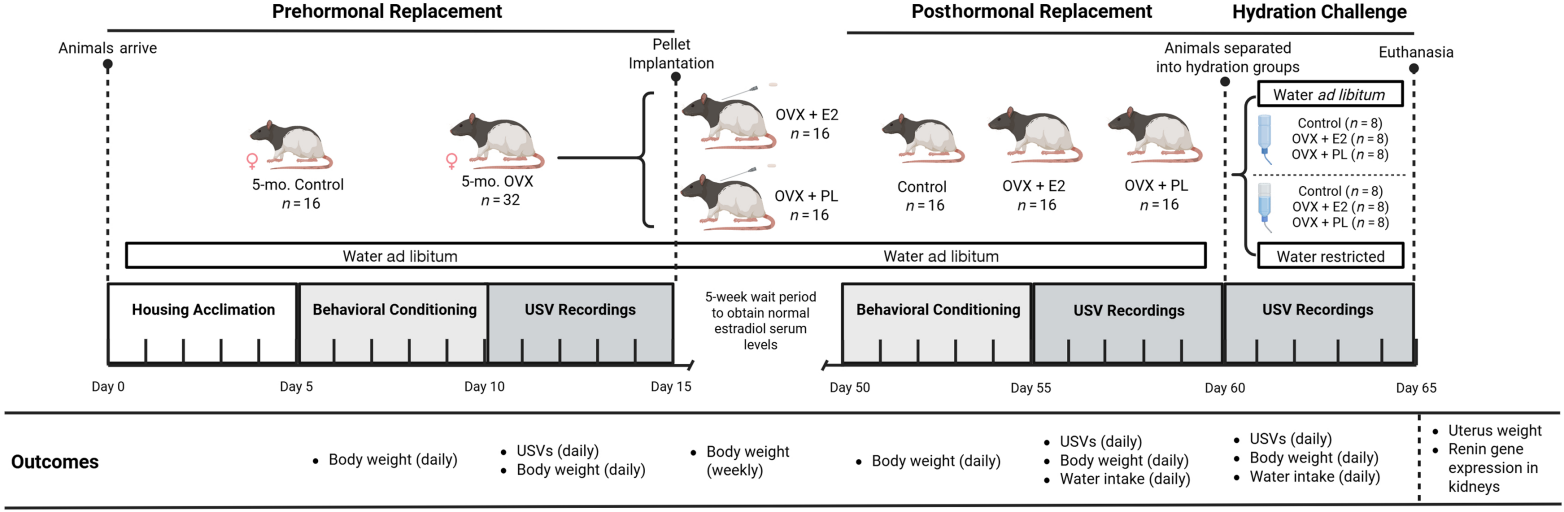
Experimental timeline demonstrating three phases of USV collection: prehormonal replacement, posthormonal replacement, and posthydration challenge (euhydration vs. dehydration). Acclimation and behavioral conditioning phases are also shown. OVX rats received E2 or PL pellets on Day 15. Final tissue collection occurred on Day 65. USV = ultrasonic vocalization; OVX = ovariectomized; E2 = estradiol; PL = placebo; mo. = months. Created in BioRender. Rodgers, B. (2026) https://BioRender.com/d10req4.

Three research questions were investigated through this study: (1) Does the USV profile differ in females with reduced circulating estrogen (OVX) as compared with controls? (2) Do OVX + Estradiol females exhibit a USV profile more similar to controls compared with OVX + Placebo females? (3) Are the USV effects of estradiol replacement different in systemic DEHY from those in systemic EUHY? The first two questions were designed to replicate and validate previously reported estrogen-related effects on USVs within the food-reward elicitation paradigm, thereby providing necessary context for testing the Estrogen × Hydration interaction posed in Question 3. We hypothesized that OVX females would produce fewer USVs, but these USVs would have a similar frequency range, power, and duration compared with those of the controls, which is consistent with prior studies showing that ovariectomy reduces 50-kHz USV number in mating contexts, without substantially altering acoustic parameters (Garcia et al., 2017; Lenell & Johnson, 2021). In contrast, OVX + Estradiol females were expected to exhibit a USV profile more similar to controls in number and acoustic features, based on evidence that estradiol replacement can partially restore USV production in some contexts, although effects are mixed and may depend on social conditions or the presence of other hormones (Garcia et al., 2017; Lenell et al., 2021). Thus, we hypothesized that OVX + Estradiol and controls would demonstrate an increased number of USVs, expanded frequency range, decreased power, and increased USV duration as compared with OVX + Placebo. Regarding hydration, we expected that EUHY would allow estradiol-driven changes in USV number to manifest fully, whereas mild DEHY would attenuate these effects by reducing tissue hydration and viscoelastic properties and potentially limiting USV number without substantially altering acoustic structure. Therefore, we further hypothesized that the presence of estradiol in euhydrated but not dehydrated animals would increase the number of USVs, expand the frequency range, decrease power, and increase USV duration. This beneficial effect would be reduced in OVX + Placebo. USVs in control rats were not expected to change significantly with mild DEHY alone, consistent with prior work showing resilience of acoustic features to minor systemic perturbations (Rodgers et al., 2025).

## Method

### Animal Model and Experimental Manipulations

This study was approved by the Purdue Animal Care and Use Committee (No. 2111002220). A total of 48 female Long–Evans rats (Envigo), aged 5 months at arrival, were used. Sixteen rats were control (intact or non-OVX), and 32 rats were OVX. The ovariectomy was performed by an accredited vendor under anesthesia before animal arrival and is considered a well-established, minimally invasive procedure (Khajuria et al., 2012; McGee-Lawrence et al., 2013; Yousefzadeh et al., 2020). Rats were allowed to recover for several weeks prior to USV recordings to ensure normal behavior and physiological stabilization (Ström et al., 2008; Theodorsson et al., 2005; Zhang et al., 2020). Upon arrival, the rats underwent an acclimation period for 5 days in single-housing conditions with enrichment toys in a temperature- and humidity-controlled facility (12-hr light/dark cycle; 6 a.m./6 p.m.). During this time, animals received daily socialization and were handled by research staff. Sample sizes were determined using a priori power considerations informed by effect sizes from our published work (Cox et al., 2021; do Nascimento et al., 2020; Duan et al., 2021) and by sample sizes commonly reported in rodent studies (Bijangi-Vishehsaraei et al., 2016; Kim, Kim, et al., 2020; Kim, Shin, et al., 2020; Lenell & Johnson, 2021; Smith et al., 2009; Welham et al., 2015).

#### USV Conditioning

During the conditioning phases, the rats were trained to vocalize within a recording chamber (SmartChamber; Metris B.V.) utilizing an established anticipatory food-reward paradigm (Brenes & Schwarting, 2015; Opiol et al., 2015). This procedure established an association between being in the chamber and receiving a treat reward immediately upon exiting, thereby encouraging the production of USVs in anticipation of the food reward. Food deprivation was not used to avoid stress-related confounds that could independently alter vocal behavior. Each morning, between 9 and 11 o'clock during the animals' light cycle, the rats were transported from the vivarium to the behavioral recording room, where they were placed in a clean, empty hard-bottomed cage without bedding inside the closed, dark chamber for 10-min recording sessions. Upon removal from the chamber, they were immediately provided with a food reward (alternating between fruit loops and veggie straws) in their home cage outside the chamber. Delivering the reward outside the chamber minimized distraction during recording while preserving anticipatory vocalization, consistent with prior validated protocols. The animals were randomly assigned to the recording chambers each day.

#### Hormonal Replacement

The 32 OVX rats were randomly assigned to receive a subcutaneous slow-release pellet containing either 0.5-mg 17β-estradiol (OVX + Estradiol) or placebo (OVX + Placebo; Innovative Research of America). Pellet implantation was performed under isoflurane anesthesia (1%–3% in oxygen at a flow rate of 1–2 L/min). Pellets were implanted 4 weeks following OVX surgery, and a 5-week recovery period followed implantation to allow estradiol levels to stabilize. This interval was determined based on pilot data (not shown) demonstrating that circulating estradiol reached normal physiological levels by 5 weeks postimplantation, as assessed via serum enzyme-linked immunosorbent assay (Estradiol Rat ELISA; ALPCO), consistent with prior validation studies (Ström et al., 2008; Theodorsson et al., 2005). The 16 control rats did not receive any hormonal manipulation. All rats had ad libitum access to dry pellet food (2018S Teklad Global 18% Protein Rodent Diet; Inotiv) and water.

#### Prehormonal Replacement USVs

USVs were recorded (described below) prior to hormonal replacement for 5 days. Body weights were also measured daily. All rats had ad libitum access to water and dry pellet food during this period.

#### Posthormonal Replacement USVs

USVs were recorded after hormonal replacement (estradiol or placebo) or control for 5 days. Body weight was measured daily during these procedures. All rats had ad libitum access to water and dry pellet food during this period.

#### Hydration Challenge

All 48 rats were then randomly assigned either to EUHY (ad libitum water) or DEHY (4-ml water/100-g body weight/day; Duan et al., 2021) for 5 days.

#### Posthydration USVs

USVs were collected daily for 5 days during the hydration challenge. Body weights were also measured daily.

#### Euthanasia

At the end of the hydration challenge, animals were euthanized with gradual-fill CO_2_ in their home cage, followed by thoracotomy. Postmortem assessments included uterus weight and kidney renin gene expression analysis (see below).

### USV Procedure

#### Recording Procedure

After behavioral conditioning, USVs were recorded utilizing the Sonotrack and SmartChamber systems (Metris B.V.). Up to four animals were simultaneously recorded in four separate chambers (one animal per chamber), each equipped with anechoic foam to prevent crosstalk. The Sonotrack system was fitted with an MIC-1 gold foil electrostatic transducer microphone (frequency range: 15–125 kHz; sensitivity: −42 dB at 50 kHz; beam angle: 12° at 3 dB down) at the top of the chamber. Animals were individually placed inside the hard-bottomed cages in the center of the sound-attenuating recording chamber and allowed to move freely within the cage during recordings. Although the estrous cycle of rodents has been reported to affect USVs, this was not measured in this study (Lenell & Johnson, 2021; Matochik et al., 1992); however, the 4- to 5-day cycle for Long–Evans rats was captured in all recording periods. Recording settings were held constant across sessions to ensure comparability between groups. All rats successfully emitted USVs within the recording chamber throughout the study.

#### Analysis Procedure

Raw USV recordings were analyzed using Sonotrack Call Classification software (Metris B.V.). Only 50-kHz USVs longer than 4 ms were included and classified as either simple (single acoustic component) or complex (multiple components) per Metris guidelines (Metris, 2017). USVs were classified as simple or complex based on established acoustic criteria (Wright et al., 2010). This distinction reflects differences in frequency modulation within USVs and has been widely used to capture functionally meaningful variation in rodent USV patterns. Acoustic variables analyzed for each USV complexity type (simple vs. complex) included total USV number, duration (in seconds), frequency range (in kilohertz), and maximum power (in dB). Sonotrack software calculates maximum signal amplitude relative to an internal reference level. These dB values reflect relative sound pressure levels (SPLs) and were used as a proxy for vocal power. The system is not calibrated to report absolute sound pressure (i.e., dB SPL); thus, all reported values should be interpreted as relative measures of vocal intensity across recording periods. Sonotrack-generated .csv files were processed using a custom R script (Version 4.3.2; R Core Team) to extract the acoustic features of interest at the USV level. Components in multicomponent (complex) USVs were grouped by a composite key (rat ID, recording day, and USV index) to summarize USV-level data. For each USV, maximum and minimum frequencies were identified across components, and the frequency range was calculated as their difference. Maximum power was the highest value among components, and total duration was taken from the first component. This structured approach allowed us to isolate acoustic features at the USV level, enabling downstream statistical analyses of how hormonal state and hydration status modulate USVs (see below). All analyses were performed on raw USV-level data, while average values per animal across recording days were calculated and used only for graphical display in the figures.

### Additional Physiological Assessments

To verify the effectiveness of hormonal manipulations and DEHY protocols beyond behavioral observations, physiological markers were evaluated, including body weight change, water intake, uterine weight, and kidney renin gene expression. Body weight was recorded at the time points described above. Body weight change relative to baseline served as a noninvasive indicator of fluid balance, with decreases interpreted as systemic DEHY as a result of reduced water intake (do Nascimento, dos Santos, et al., 2022; Duan et al., 2021; Islam et al., 2004; King et al., 2018; Rodgers et al., 2025; Schwarz et al., 2010). During the hydration challenge, daily water intake was measured by recording changes in water bottle volume in each cage, with adjustments for evaporation or typical loss based on a control bottle. Average daily water intake and body weight were compared across groups to confirm the systemic effects of DEHY.

At euthanasia, uterine weight was collected, and kidney cortex tissue was harvested for renin gene expression. Uterine weight was collected as a well-established surrogate marker to confirm systemic estrogen status following ovariectomy. Loss of circulating estrogen reliably results in uterine atrophy and reduced uterine mass, whereas estradiol replacement restores uterine structure and weight toward intact levels (Behr et al., 2012; Gogos et al., 2018; Høegh-Andersen et al., 2004; Li et al., 2014; Smith et al., 2009; Zhang et al., 2020). Accordingly, uterine weight was included to verify the effectiveness of estrogen manipulation independent of hydration status. Kidney cortex tissues were preserved in RNAlater Stabilization Solution (Invitrogen). Total RNA was extracted using the RNeasy Plus Mini Kit (QIAGEN) and reverse transcribed into cDNA using SuperScript IV VILO Master Mix (Invitrogen). Renin (*Ren*) gene expression was quantified by reverse transcription–quantitative polymerase chain reaction (PCR) using previously validated primers for rat tissue with β-actin (*Actb*) as the normalizer gene (Venkatraman et al., 2022). Reactions were performed on a QuantStudio 3 Real-Time PCR System (Applied Biosystems) using standard cycling conditions. Relative expression was calculated via the 2^−ΔΔCt^ method (Livak & Schmittgen, 2001), and *Ren* gene expression was assessed as an exploratory renal renin–angiotensin–aldosterone system (RAAS)–related molecular outcome. Primary validation of hydration manipulation in dehydrated animals was based on reduced water intake and DEHY-associated body weight loss.

### Statistical Analysis

All statistical analyses were conducted in R (Version 4.3.2). The analyses were selected after detailed consultations with a biostatistician (author J.X.). Generalized linear mixed-effects models were used to examine the effects of hormonal and hydration manipulations on USV outcomes. Separate models were fit to raw data for each acoustic measure (total number, duration, frequency range, maximum power) by USV complexity type (simple, complex), with fixed effects varying by research question. Comparisons included hormonal group (OVX + Estradiol, OVX + Placebo, control), hydration status (EUHY, DEHY), time (prehormonal replacement, posthormonal replacement, posthydration challenge), and their interactions. Random intercepts were included for animal ID nested within a cohort to account for repeated measures and batch-level variation. Model families were selected based on outcome distribution: gamma models for strictly positive and right-skewed duration and frequency range data, linear mixed-effects models for normally distributed maximum power, and negative binomial models for USV total number. Model assumptions and fit were assessed using residual diagnostics and information criteria. Model effects were evaluated using Type III Wald χ^2^ tests. Significant effects were followed by planned contrasts using estimated marginal means, restricted to interactions and main effects directly addressing the study's a priori research questions, with Tukey's correction for multiple comparisons. Physiological outcomes (body weight and water intake change, uterine weight, and renin gene expression) were analyzed in GraphPad Prism 10 using Welch's *t* tests or two-way analyses of variance (ANOVAs), as appropriate. Main effects and interactions of hormonal group and hydration status were evaluated, with post hoc Tukey's or Šídák's multiple comparisons tests conducted for significant effects. All significant results are reported with effect sizes (η^2^ or Cohen's *d*, where appropriate). Significance was set at *p* ≤ .05.

## Results

Forty-eight female Long–Evans rats were distributed across six experimental groups (*n* = 8 per group) defined by hormonal state and hydration status: EUHY control, DEHY control, EUHY OVX + Estradiol, DEHY OVX + Estradiol, EUHY OVX + Placebo, and DEHY OVX + Placebo. Across all time points, a total of 102,547 USVs were recorded (82,893 simple and 19,654 complex). All rats emitted USVs on all days of recording, except for one EUHY OVX + Estradiol animal, which did not vocalize on the final day of EUHY. Model outcomes indicated that hormonal and hydration status significantly affected multiple USV features. The statistically significant results for each research question are reported below.

### USV Profile of Reduced Circulating Estrogen (OVX) Versus Controls

**Figure 2.**
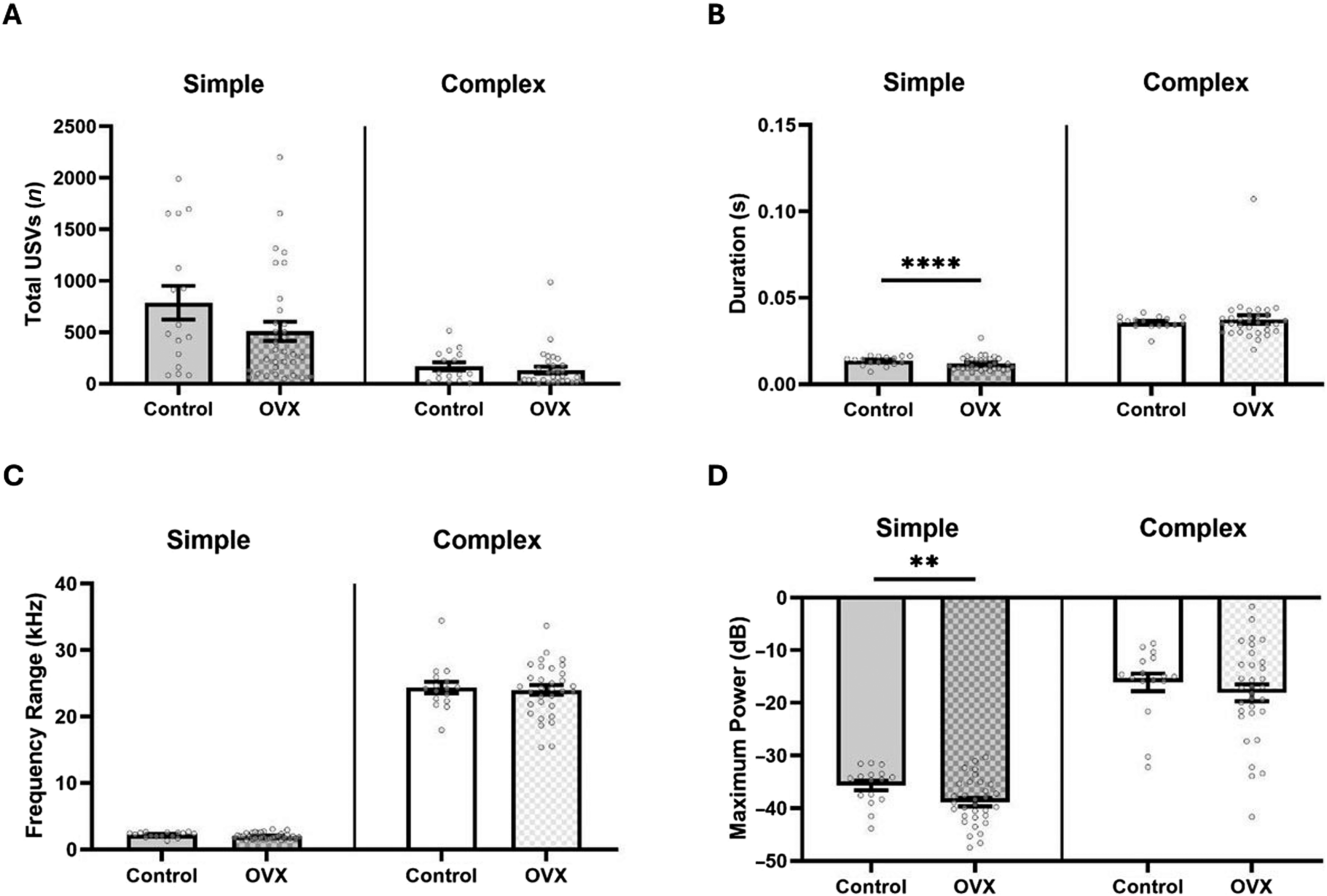
Prehormonal replacement USVs for OVX (*n* = 32) versus controls (*n* = 16). Total USV number (A), USV duration (B), USV frequency range (C), and USV maximum power (D). Bar graphs represent group means ± *SEM*s, and individual data points represent measurements from each animal within each group. Bar fill patterns indicate hormonal group: solid = controls and cross-hatched = OVX. **p < .01, ****p < .0001. USVs = ultrasonic vocalizations; OVX = ovariectomized.

OVX females exhibited selective USV changes relative to controls. The means ± standard errors of the mean (*SEM*s) for prehormonal replacement USVs are shown in Table 1. Significant Time × Group interactions were observed for simple total number (*p* = .01), simple duration (*p* < .001), and simple power (*p* < .001; see Table 2). Post hoc comparisons indicated that simple duration was shorter (*p* < .0001, *d* = 0.09) and simple power was lower (*p* = .007, *d* = 0.13) in OVX animals (see Figure 2 and Table 3). There was a trend toward fewer simple USVs for OVX animals (*p* = .06, *d* = 0.25). Together, these findings suggest that estrogen reduction primarily affects temporal and amplitude aspects of USVs rather than their quantity or spectral breadth.

**Table 1.** Estimated marginal means ± *SEM*s for ultrasonic vocalization (USV) measures in each group and time point.

Prehormonal replacement USVs	Controls	OVX
Simple total number	103.5 ± 36.7	65.9 ± 21.5
Simple duration (s)	0.0132 ± 9.97e-05	0.0113 ± 5.98e-05
Simple frequency range (kHz)	2.22 ± 0.145	2.03 ± 0.118
Simple power (dB)	−35.5 ± 1.52	−38.5 ± 1.39
Complex total number	20.9 ± 8.73	15.2 ± 5.89
Complex duration (s)	0.0350 ± 0.00189	0.0351 ± 0.00162
Complex frequency range (kHz)	23.7 ± 0.820	24.2 ± 0.629
Complex power (dB)	−15.7 ± 2.72	−17.8 ± 2.5

Posthormonal replacement USVs	Controls	OVX + Estradiol	OVX + Placebo
Simple total number	109.1 ± 38.7	42.7 ± 15.2	63.0 ± 22.4
Simple duration (s)	0.0136 ± 1.06e-04	0.0106 ± 7.18e-05	0.0116 ± 1.01e-04
Simple frequency range (kHz)	2.34 ± 0.153	1.83 ± 0.121	2.05 ± 0.134
Simple power (dB)	−34.5 ± 1.52	−40.3 ± 1.56	−38.3 ± 1.54
Complex total number	23.7 ± 9.90	10.6 ± 4.44	14.2 ± 5.96
Complex duration (s)	0.0355 ± 0.00193	0.0333 ± 0.00169	0.0357 ± 0.00198
Complex frequency range (kHz)	23.7 ± 0.819	24.3 ± 0.908	24.7 ± 0.897
Complex power (dB)	−15.4 ± 2.72	−20.2 ± 2.77	−17.2 ± 2.76

Posthydration challenge USVs	EUHY controls	DEHY controls	EUHY OVX + Estradiol	DEHY OVX + Estradiol	EUHY OVX + Placebo	DEHY OVX + Placebo
Simple total number	82.3 ± 29.3	131.8 ± 53.1	41.3 ± 14.7	69.5 ± 24.7	99.4 ± 35.4	76.1 ± 27.1
Simple duration (s)	0.01385 ± 1.57e-04	0.01269 ± 1.45e-04	0.00927 ± 9.71e-05	0.00983 ± 9.95e-05	0.01391 ± 2.61e-04	0.01130 ± 1.40e-04
Simple frequency range (kHz)	2.32 ± 0.181	2.02 ± 0.157	1.75 ± 0.137	1.80 ± 0.140	2.20 ± 0.172	1.93 ± 0.151
Simple power (dB)	−35.4 ± 2.04	−37.7 ± 2.03	−41.8 ± 2.08	−41.0 ± 2.05	−37.8 ± 2.04	−38.4 ± 2.05
Complex total number	20.38 ± 14.68	25.32 ± 10.70	8.69 ± 3.69	14.31 ± 6.06	27.35 ± 27.35	13.89 ± 5.89
Complex duration (s)	0.0351 ± 0.00211	0.0363 ± 0.00229	0.0305 ± 0.00160	0.0322 ± 0.00177	0.0381 ± 0.0400	0.0350 ± 0.00217
Complex frequency range (kHz)	24.8 ± 1.33	23.9 ± 1.24	25.8 ± 1.48	25.8 ± 1.39	22.2 ± 1.18	23.1 ± 1.26
Complex power (dB)	−18.1 ± 3.36	−16.3 ± 3.31	−19.7 ± 3.45	−20.6 ± 3.36	−18.7 ± 3.34	−19.1 ± 3.37

*Note.* OVX = ovariectomized; EUHY = euhydration; DEHY = dehydration.

**Table 2.** Generalized linear mixed-effect model results for prethormonal and posthormonal replacement ultrasonic vocalizations (USVs).

USV outcome	Fixed effect	χ^2^	*df*	*p* value	Significance
Simple total number	Time	4.0174	1	.04503	*
	Group	7.3082	2	.02589	*
	Time × Group	8.6052	2	.01353	*
Simple duration (s)	Time	0.6655	1	.4146	
	Group	1,331.7300	2	< .0001	***
	Time × Group	94.1371	2	< .0001	***
Simple frequency range (kHz)	Time	4.0814	1	.04336	*
	Group	7.7039	2	.02124	*
	Time × Group	97.3569	2	< .0001	***
Simple power (dB)	Time	0.6477	1	.4209306	
	Group	13.9817	2	.0009203	***
	Time × Group	18.4784	2	< .0001	***
Complex total number	Time	1.4898	1	.22225	
	Group	3.7380	2	.15428	
	Time × Group	8.6050	2	.01354	*
Complex duration (s)	Time	0.8026	1	.3703	
	Group	0.3121	2	.8555	
	Time × Group	20.3980	2	< .0001	***
Complex frequency range (kHz)	Time	0.5336	1	.4651	
	Group	1.3618	2	.5062	
	Time × Group	2.7959	2	.2471	
Complex power (dB)	Time	0.8709	1	.3507	
	Group	2.6061	2	.2717	
	Time × Group	4.5385	2	.1034	

*Note.* Single generalized linear mixed-effect model was used, including time (prehormonal vs. posthormonal replacement) and hormonal group (controls, OVX + Estradiol, OVX + Placebo). Model effects were evaluated using Type III Wald χ^2^ tests. OVX = ovariectomized.

* *p* < .05.

*** *p* < .001.

**Table 3.** Post hoc pairwise comparisons after significant effects in the generalized linear mixed-effect model for prehormonal and posthormonal replacement ultrasonic vocalizations (USVs).

USV outcome	Prehormonal replacement USVs			Posthormonal replacement USVs		
	Post hoc contrast	*z*	*p* value	Post hoc contrast	*z*	*p* value
Simple total number	Controls vs. OVX	1.868	.0617	Controls vs. OVX + Estradiol	3.361	**.0022**
				Controls vs. OVX + Placebo	1.973	.1188
				OVX + Estradiol vs. OVX + Placebo	−1.389	.3466
Simple duration (s)	Controls vs. OVX	−20.343	**< .0001**	Controls vs. OVX + Estradiol	−33.367	**< .0001**
				Controls vs. OVX + Placebo	−14.074	**< .0001**
				OVX + Estradiol vs. OVX + Placebo	7.537	**< .0001**
Simple frequency range (kHz)	Controls vs. OVX	1.71	.0872	Controls vs. OVX + Estradiol	4.042	**.0002**
				Controls vs. OVX + Placebo	2.205	.0704
				OVX + Estradiol vs. OVX + Placebo	−1.836	.1579
Simple power (dB)	Controls vs. OVX	2.692	**.0071**	Controls vs. OVX + Estradiol	4.462	**< .0001**
				Controls vs. OVX + Placebo	2.919	**.0098**
				OVX + Estradiol vs. OVX + Placebo	−1.559	.2636
Complex total number	Controls vs. OVX	1.204	.2288	Controls vs. OVX + Estradiol	2.572	**.0273**
				Controls vs. OVX + Placebo	1.636	.2303
				OVX + Estradiol vs. OVX + Placebo	−0.939	.6154
Complex duration (s)	Controls vs. OVX	0.004	.9971	Controls vs. OVX + Estradiol	−1.202	.4518
				Controls vs. OVX + Placebo	0.096	.9950
				OVX + Estradiol vs. OVX + Placebo	1.275	.4092

*Note. p* values are Tukey adjusted for multiple comparisons. Bolded values indicate significance at ≤ .05. OVX = ovariectomized (rats).

### USV Profile of OVX With Estradiol Replacement Versus Placebo and Controls

**Figure 3.**
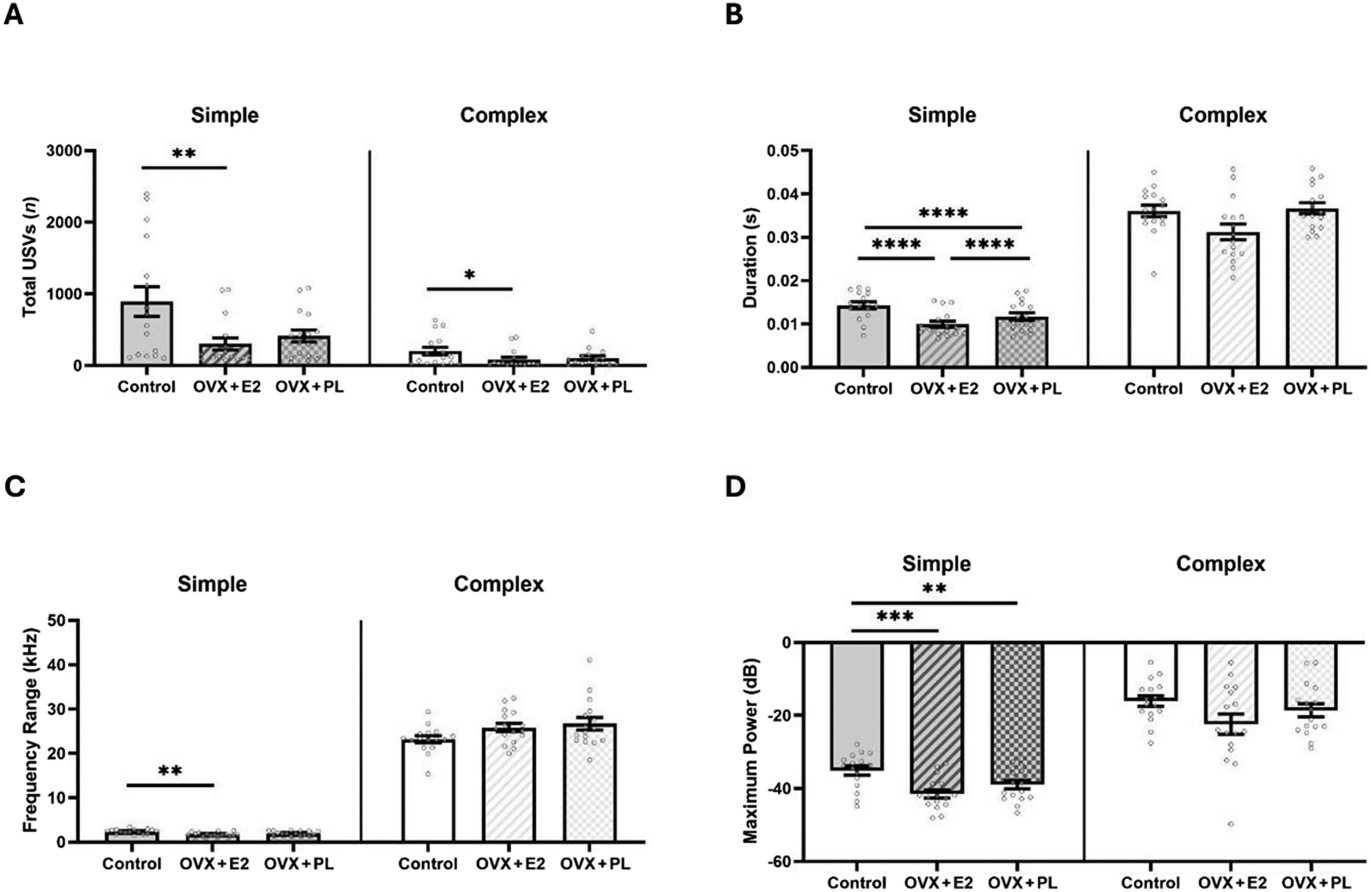
Posthormonal replacement USVs for OVX + E2 (*n* = 16), OVX + Placebo (OVX + PL; *n* = 16), and controls (*n* = 16). Total USV number (A), USV duration (B), USV frequency range (C), and USV maximum power (D). Bar graphs represent group means ± *SEM*s, and individual data points represent measurements from each animal within each group. Bar fill patterns indicate hormonal group: solid = controls, slashed = OVX + E2, and cross-hatched = OVX + PL. *p < .05, **p < .01, ***p < .001, ****p < .0001. USVs = estradiol; OVX = ovariectomized.

Significant group differences persisted after estradiol replacement for simple duration, power, total number, and frequency range and for complex total number. Means ± *SEM*s for posthormonal replacement USVs are shown in Table 1. Significant Time × Group interactions were observed for simple total number (*p* = .01; see Table 2), simple duration (*p* < .001), simple power (*p* < .001), simple frequency range (*p* < .001), and complex total number (*p* = .01). Simple duration was shorter in OVX + Estradiol (*p* < .0001, *d* = 0.21; see Table 3) and OVX + Placebo (*p* < .0001, *d* = 0.14) animals relative to controls and in OVX + Estradiol compared with OVX + Placebo animals (*p* < .0001, *d* = 0.06; see Figure 3B). Simple power was lower in OVX + Estradiol animals (*p* < .0001, *d* = 0.21) and OVX + Placebo animals (*p* = .01, *d* = 0.17) compared with controls (see Figure 3D). OVX + Estradiol animals emitted fewer simple (*p* = .002, *d* = 0.82) and complex (*p* = .03, *d* = 0.55) USVs relative to controls (see Figure 3A). OVX + Estradiol animals' simple USVs also exhibited a reduced frequency range (*p* = .0002, *d* = 0.19) compared with controls (see Figure 3C). Collectively, estradiol replacement modulated select acoustic features but did not restore a control-like profile; OVX + Estradiol animals instead demonstrated reduced USV duration, frequency, and power features.

### USV Profile Across Hormonal Status After Hydration Challenge

**Figure 4.**
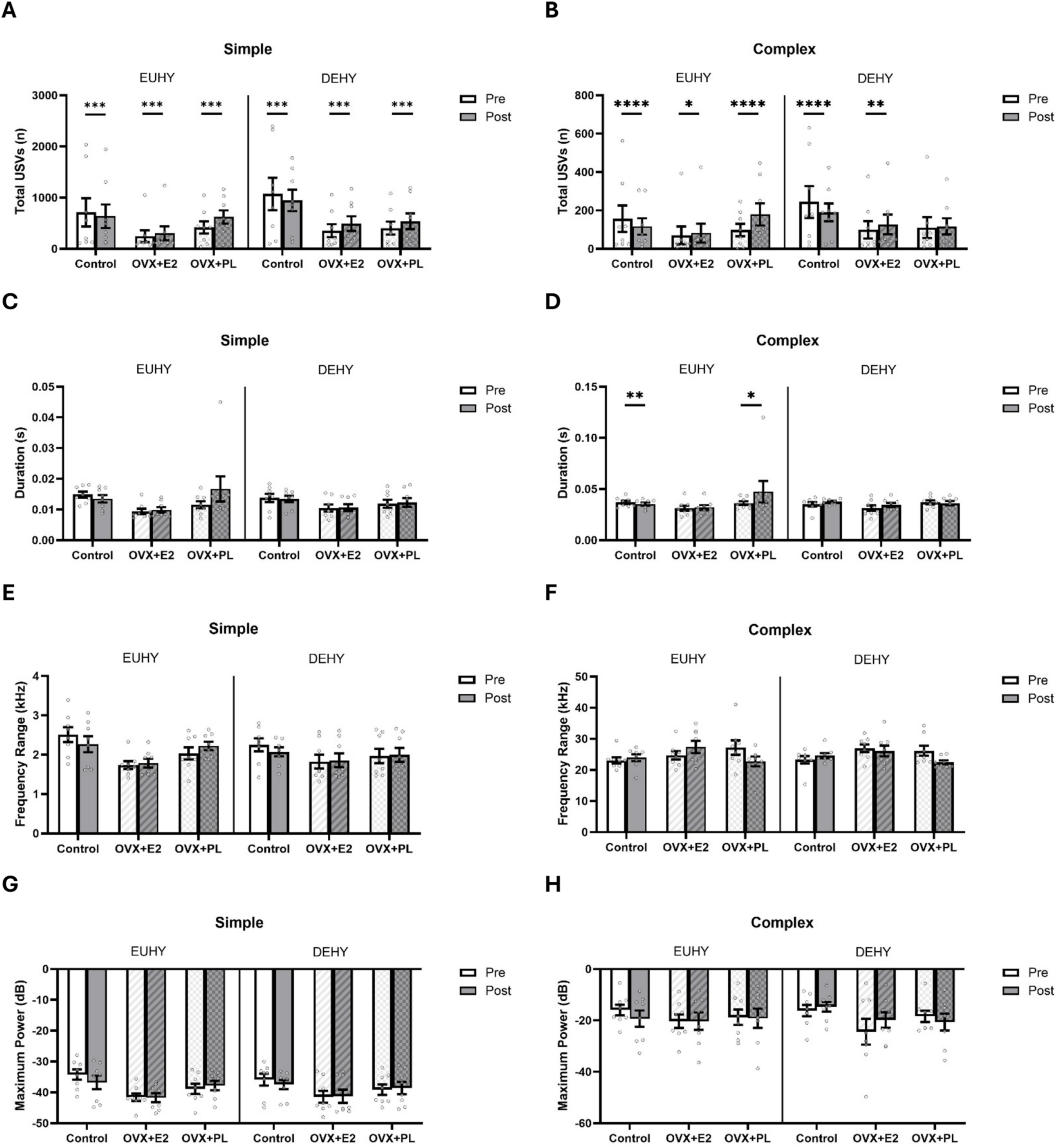
USV measures prehydration and posthydration challenge across three hormonal groups (control, OVX + E2, OVX + PL) and two hydration groups (EUHY and DEHY). *n* = 8 per group. Simple (left column) and complex (right column) measures are depicted. Total USV number (A and B), USV duration (C and D), USV frequency range (E and F), and USV maximum power (G and H). Bar graphs represent group means ± *SEM*s, and individual data points represent measurements from each animal within each group. Bar fill patterns indicate hormonal group: solid = controls, slashed = OVX + E2, and cross-hatched = OVX + PL. *p < .05, **p < .01, ***p < .001. USV = ultrasonic vocalization; OVX = ovariectomized; E2 = estradiol; PL = placebo; EUHY = euhydration; DEHY = dehydration; Pre = posthormone replacement USVs; Post = posthydration challenge USVs.

Means ± *SEM*s for posthydration challenge USVs are shown in Table 1. Significant Time × Hydration × Group interactions were found for simple total number (*p* < .001; see Table 4), complex total number (*p* < .001), and complex duration (*p* < .001). Post hoc comparisons indicated that OVX + Estradiol animals produced more simple and complex USVs post-EUHY (simple: *p* < .0001, *d* = 0.15; complex: *p* = .03, *d* = 0.04; see Table 5) and DEHY (simple: *p* < .0001, *d* = 0.27; complex: *p* = .006, *d* = 0.08; see Figures 4A and 4B). Similarly, OVX + Placebo animals increased simple and complex USV total numbers post-EUHY (simple: *p* < .0001, *d* = 0.31; complex: *p* < .0001, *d* = 0.32) and simple USV total number post-DEHY (*p* < .0001, *d* = 0.29; see Figures 4A and 4B). In contrast, controls produced fewer simple and complex USVs after EUHY (simple: *p* < .0001, *d* = 0.09; complex: *p* < .0001, *d* = 0.26) and DEHY (simple: *p* < .0001, *d* = 0.15; complex: *p* < .0001, *d* = 0.29; see Figures 4A and 4B). Complex USVs were shortened in EUHY controls (*p* = .002, *d* = 0.17) and lengthened for EUHY OVX + Placebo animals (*p* = .03, *d* = 0.53; see Figure 4D).

**Table 4.** Generalized linear mixed-effect model results for posthydration challenge ultrasonic vocalizations (USVs).

USV outcome	Fixed effect	χ^2^	*df*	*p* value	Significance
Simple total number	Time	308.4983	1	< .0001	***
	Hydration	1.4495	1	.228612	
	Group	12.1532	2	.002296	**
	Time × Hydration	0.0164	1	.897954	
	Time × Group	608.2202	1	< .0001	***
	Hydration × Group	2.5363	2	.281350	
	Time × Hydration × Group	20.3927	2	< .0001	***
Simple duration (s)	Time	0.9457	1	.33082	
	Hydration	42.5780	1	< .0001	***
	Group	2,175.8522	2	< .0001	***
	Time × Hydration	2.9540	1	.08567	
	Time × Group	54.5770	1	< .0001	***
	Hydration × Group	117.0426	2	< .0001	***
	Time × Hydration × Group	4.0506	2	.13195	
Simple frequency range (kHz)	Time	12.3337	1	.0004449	***
	Hydration	2.0149	1	.1557665	
	Group	20.4251	2	< .0001	***
	Time × Hydration	6.6035	1	.0101779	*
	Time × Group	68.5621	1	< .0001	***
	Hydration × Group	2.3213	2	.3132838	
	Time × Hydration × Group	1.5682	2	.4565302	
Simple power (dB)	Time	5.1582	1	.02314	*
	Hydration	0.4230	1	.51546	
	Group	18.7234	2	< .0001	***
	Time × Hydration	0.0180	1	.89325	
	Time × Group	29.4128	1	< .0001	***
	Hydration × Group	0.6229	2	.73238	
	Time × Hydration × Group	4.4374	2	.10875	
Complex total number	Time	7.8353	1	.005124	**
	Hydration	0.8223	1	.364518	
	Group	7.1652	2	.027803	*
	Time × Hydration	22.7607	1	< .0001	***
	Time × Group	236.8429	1	< .0001	***
	Hydration × Group	3.1702	2	.204928	
	Time × Hydration × Group	67.0567	2	< .0001	***
Complex duration (s)	Time	0.0125	1	.911145	
	Hydration	0.1393	1	.708985	
	Group	13.3282	2	.001276	**
	Time × Hydration	0.6091	1	.435143	
	Time × Group	4.4629	1	.107370	
	Hydration × Group	1.8307	2	.400385	
	Time × Hydration × Group	13.2668	2	.001316	**
Complex frequency range (kHz)	Time	0.0146	1	.90397	
	Hydration	0.6160	1	.43254	
	Group	3.7647	2	.15223	
	Time × Hydration	6.2258	1	.01259	*
	Time × Group	7.3952	1	.02478	*
	Hydration × Group	0.1186	2	.94240	
	Time × Hydration × Group	2.1459	2	.34199	
Complex power (dB)	Time	7.1147	1	.0076455	**
	Hydration	0.0005	1	.9824523	
	Group	4.9101	2	.0858604	
	Time × Hydration	0.0744	1	.7851017	
	Time × Group	15.6046	1	.0004088	***
	Hydration × Group	0.1894	2	.9096443	
	Time × Hydration × Group	1.0310	2	.5972020	

*Note.* Single generalized linear mixed-effect model was used, including time (prehydration vs. posthydration challenge), hydration (EUHY, DEHY), and hormonal group (controls, OVX + Estradiol, OVX + Placebo). Model effects were evaluated using Type III Wald χ^2^ tests. EUHY = euhydration; DEHY = dehydration; OVX = ovariectomized (rats).

* *p* < .05.

** *p* < .01.

*** *p* < .001.

**Table 5.** Post hoc pairwise comparisons after significant effects in the generalized linear mixed-effect model for posthydration ultrasonic vocalizations (USVs).

Time × Hydration × Group					
USV outcome	Post hoc contrast	Hydration	Hormonal group	*z*	*p* value
Simple total number	Prehydration vs. posthydration challenge	EUHY	Controls	5.765	**< .0001**
			OVX + Estradiol	−7.026	**< .0001**
			OVX + Placebo	−17.781	**< .0001**
		DEHY	Controls	7.898	**< .0001**
			OVX + Estradiol	−13.405	**< .0001**
			OVX + Placebo	−12.320	**< .0001**
Complex duration (s)	Prehydration vs. posthydration challenge	EUHY	Controls	−3.103	**.0019**
			OVX + Estradiol	0.095	.9247
			OVX + Placebo	2.151	**.0315**
		DEHY	Controls	1.063	.2878
			OVX + Estradiol	1.069	.2850
			OVX + Placebo	−0.850	.3955
Complex total number	Prehydration vs. posthydration challenge	EUHY	Controls	7.568	**< .0001**
			OVX + Estradiol	−2.113	**.0346**
			OVX + Placebo	−14.270	**< .0001**
		DEHY	Controls	8.024	**< .0001**
			OVX + Estradiol	−2.770	**.0056**
			OVX + Placebo	−0.677	.4986

Time × Hydration				
USV outcome	Post hoc contrast	Hydration	*z*	*p* value
Simple frequency range (kHz)	Prehydration vs. posthydration challenge	EUHY	0.622	.5336
		DEHY	4.652	**< .0001**
Complex frequency range (kHz)	Prehydration vs. posthydration challenge	EUHY	−1.726	.0843
		DEHY	1.819	.0689

Group main effect			
USV outcome	Post hoc contrast	*z*	*p* value
Simple duration (s)	Controls vs. OVX + Estradiol	−45.608	**< .0001**
	Controls vs. OVX + Placebo	−8.424	**< .0001**
	OVX + Estradiol vs. OVX + Placebo	23.500	**< .0001**
Simple power (dB)	Controls vs. OVX + Estradiol	4.327	**< .0001**
	Controls vs. OVX + Placebo	2.123	.0853
	OVX + Estradiol vs. OVX + Placebo	−2.209	.0697

*Note.* Post hoc analyses were completed for significant Time × Hydration × Group and Time × Hydration interactions, as well as group main effect, which directly relate to the study's research questions. *p* values are Tukey adjusted for multiple comparisons. Bolded values indicate significance at ≤ .05. EUHY = euhydration; OVX = ovariectomized (rats); DEHY = dehydration.

**Figure 5.**
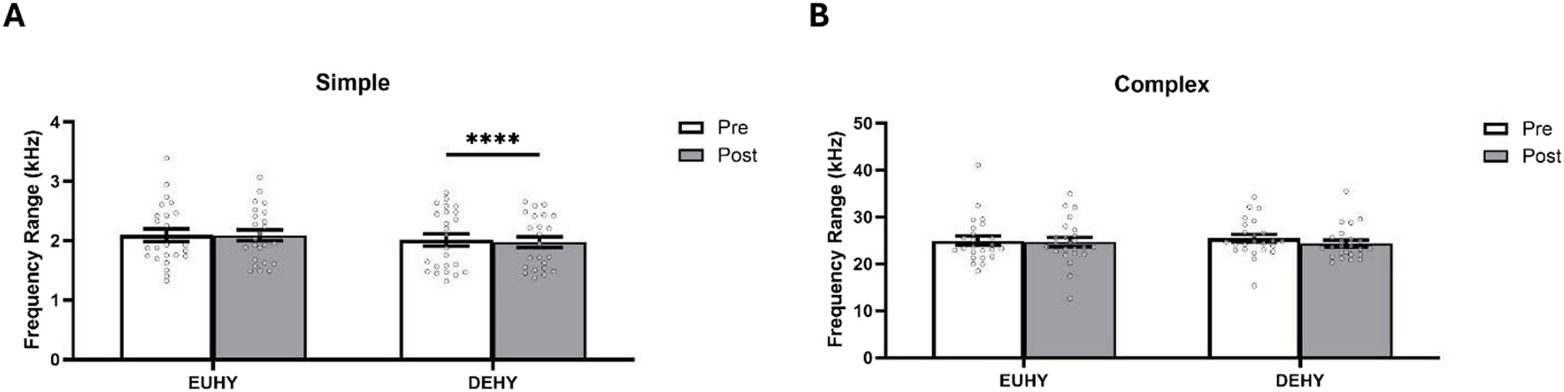
Time × Hydration interaction for USV frequency range across two hydration groups (EUHY and DEHY). *n* = 24 per group. Simple (A) and complex (B) measures are depicted. Bar graphs represent group means ± *SEM*s stratified across hormonal group, and individual data points represent measurements from each animal within each hydration group. ****p < .0001. USV = ultrasonic vocalization; EUHY = euhydration; DEHY = dehydration; Pre = posthormone replacement USVs; Post = posthydration challenge USVs.

Additional Time × Hydration interactions were detected for simple frequency range (*p* = .01; see Table 4) and complex frequency range (*p* = .01). Hydration status, independent of hormonal group, significantly reduced the frequency range of simple USVs in DEHY animals (*p* < .0001, *d* = 0.01; see Figure 5A and Table 5). A similar reduction in complex frequency range at the trend level was found in DEHY animals (*p* = .07; see Figure 5B).

**Figure 6.**
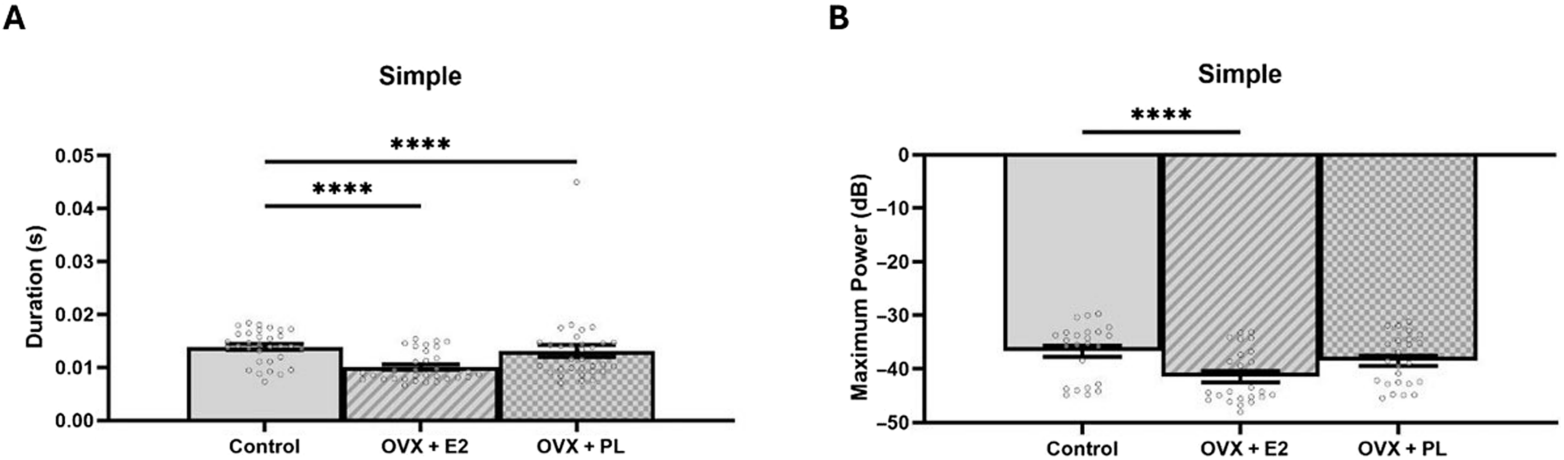
Hormonal group main effects for simple USV duration (A) and maximum power (B) across three hormonal groups (control, OVX + E2, OVX + PL). *n* = 16 per group. Bar graphs represent group means ± *SEM*s stratified across time (posthormone replacement and posthydration challenge USVs) and hydration group, and individual data points represent measurements from each animal within each hormonal group and both time points. Bar fill patterns indicate hormonal group: solid = controls, slashed = OVX + E2, and cross-hatched = OVX + PL. ****p < .0001. USV = ultrasonic vocalization; OVX = ovariectomized; E2 = estradiol; PL = placebo.

Hormonal group main effects were present for simple duration (*p* < .001; see Table 4) and simple power (*p* < .001), as indicated before, and remained regardless of hydration condition. That is, simple duration was shorter in OVX + Estradiol (*p* < .0001, *d* = 0.01; see Table 5) and OVX + Placebo (*p* < .0001, *d* = 0.01) animals compared with controls (see Figure 6A). Simple power was lower in OVX + Estradiol animals (*p* < .0001, *d* = 0.56) and at a trend level in OVX + Placebo animals (*p* = .09, *d* = 0.16) compared with controls (see Figure 6B). Overall, estradiol increased simple and complex USV numbers in OVX animals across hydration conditions, whereas controls showed reductions in USV number following hydration challenges. DEHY broadly reduced frequency range across hormonal groups, and simple USVs were shorter and lower in power in OVX + Estradiol animals, indicating that hormone status and hydration interact to shape both the quantity and temporal–spectral features of USVs.

### Physiological Validation

Means ± standard deviations for physiological measures, including percent body weight and water intake change, renin gene expression, and uterine weight, are shown in Table 6.

**Table 6.** Physiological validation of hormonal state and hydration status.

Prehormonal replacement	Controls	OVX
Body weight (g)	272.5 ± 13.7	292.8 ± 26.2

Posthormonal replacement	Controls	OVX + Estradiol	OVX + Placebo
Body weight (g)	280.4 ± 18.7	288.9 ± 31.1	329.5 ± 27.1

Posthydration challenge	EUHY controls	DEHY controls	EUHY OVX + Estradiol	DEHY OVX + Estradiol	EUHY OVX + Placebo	DEHY OVX + Placebo
Body weight change (%)	0.4 ± 1.5	−6.0 ± 1.1	1.0 ± 1.4	−7.9 ± 2.5	0.1 ± 1.0	−3.5 ± 1.2
Water intake change (%)	8.5 ± 25.5	−59.5 ± 9.7	−4.1 ± 8.3	−69.8 ± 2.0	−0.6 ± 9.9	−46.0 ± 3.7
Relative renin expression	1.3 ± 1.1	1.7 ± 0.7	1.1 ± 0.5	0.7 ± 0.3	1.2 ± 0.9	1.8 ± 0.9
Uterine weight (g)	0.6 ± 0.1	0.6 ± 0.3	0.3 ± 0.1	0.2 ± 0.1	0.1 ± 0.0	0.1 ± 0.0

*Note.* Values are reported as means ± *SD*s for each group and time point. OVX = ovariectomized (rats); EUHY = euhydration; DEHY = dehydration.

#### Body Weight

**Figure 7.**
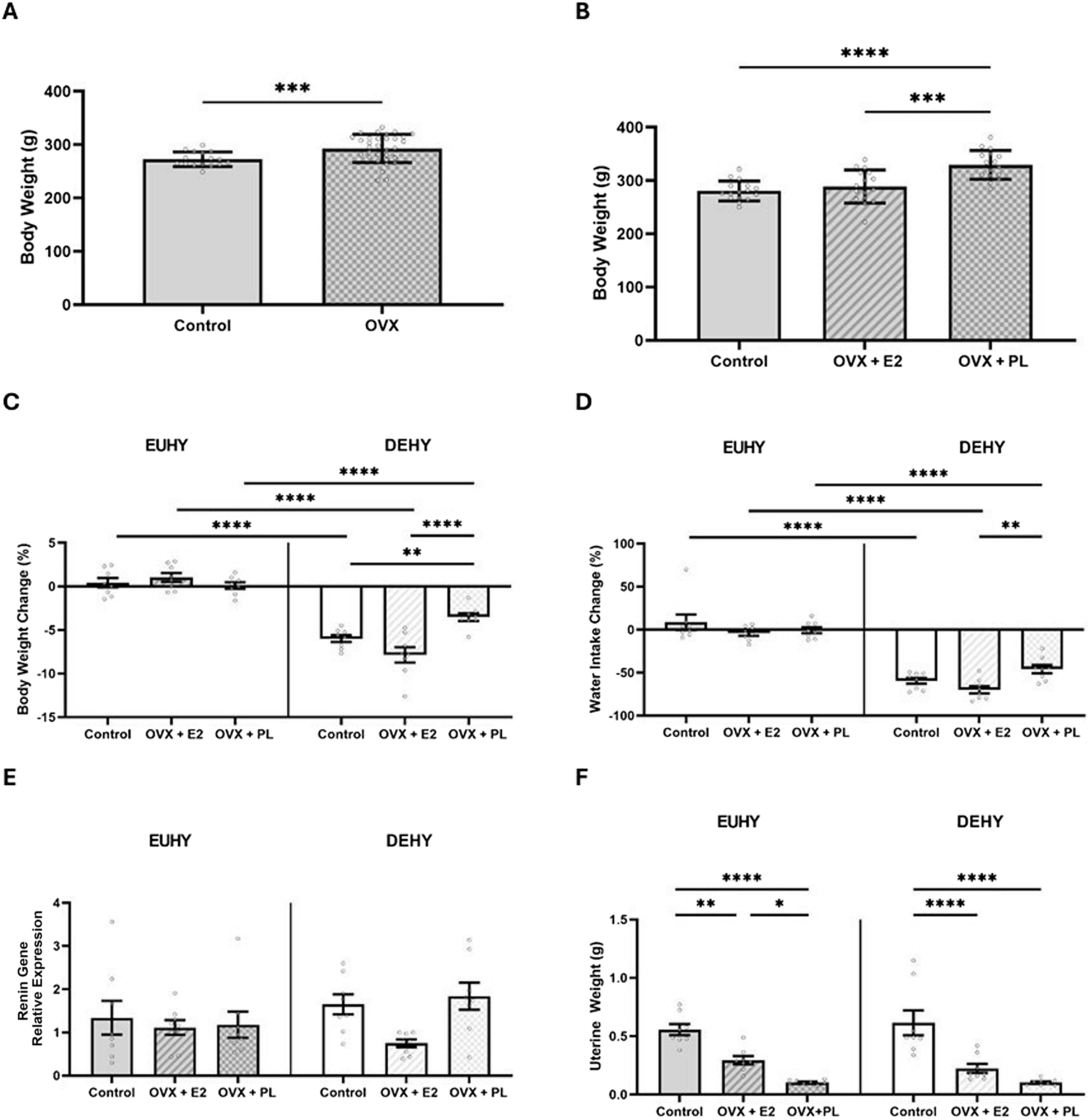
Physiological validation of hormonal state and hydration status. (A and B) Body weight across the prehormonal and posthormonal replacement phases by hormonal group (control, OVX + E2, OVX + PL). (C) Percent body weight change after the hydration challenge by group. (D) Percent water intake change during the hydration challenge by group. (E) Renin gene relative expression in kidney tissue for each group. (F) Uterine weight at tissue collection for each group. Bar graphs represent group means ± *SD*s, and individual data points represent measurements from each animal within each group. Bar fill patterns indicate hormonal group: solid = controls, slashed = OVX + E2, and cross-hatched = OVX + PL. *p < .05, **p < .01, ***p < .001, ****p < .0001. OVX = ovariectomized; E2 = estradiol; PL = placebo; EUHY = euhydration; DEHY = dehydration.

Body weight at prehormonal replacement was significantly higher in OVX rats compared with controls, Welch's *t*(45.85) = 3.54, *p* = .0009, η^2^ = .21 (see Figure 7A). A one-way ANOVA revealed a significant effect of hormonal group on posthormonal replacement body weight, *F*(2, 45) = 16.15, *p* < .001, η^2^ = .42. Tukey's post hoc comparisons showed that OVX + Placebo animals weighed significantly more than both OVX + Estradiol (*p* = .0002) and control (*p* < .001) animals (see Figure 7B). A two-way ANOVA was conducted on the percent body weight change after the hydration challenge. There was a significant interaction between hormonal group and hydration status, *F*(2, 42) = 11.63, *p* < .001, η^2^ = .20, indicating that the effect of hydration differed across hormonal groups. Both main effects were also significant: group, *F*(2, 42) = 4.93, *p* = .012, η^2^ = .11, and hydration status, *F*(1, 42) = 199.0, *p* < .001, η^2^ = .73. On average, EUHY rats gained weight, whereas DEHY rats lost weight (see Figure 7C). Tukey's simple-effects comparisons showed no significant group differences under EUHY (all *p*s > .45). Under DEHY, OVX + Placebo rats lost significantly less weight than both controls (*p* = .007) and OVX + Estradiol rats (*p* < .001; see Figure 7C). The difference between controls and OVX + Estradiol was not significant (*p* = .053).

#### Water Intake

A two-way ANOVA was conducted on the percent water intake change during the hydration challenge. The interaction between hormonal group and hydration status was not significant, *F*(2, 42) = 3.00, *p* = .061, η^2^ = .05. There were a significant main effect of group, *F*(2, 42) = 4.15, *p* = .023, η^2^ = .09, and a robust main effect of hydration status, *F*(1, 42) = 206.1, *p* < .001, η^2^ = .79. On average, EUHY rats increased water intake slightly, whereas DEHY rats showed a substantial reduction (see Figure 7D). Post hoc Tukey's comparisons for the group main effect indicated that OVX + Estradiol rats decreased water intake more than OVX + Placebo rats did (see Figure 7D). Differences between controls and OVX + Estradiol or OVX + Placebo animals were not significant (*p* = .074 and *p* = .904, respectively).

#### Renin Gene Expression

A two-way ANOVA was conducted on renin gene relative expression, with hormonal group and hydration status as factors. The main effect of hormonal group trended toward significance, *F*(2, 44) = 2.90, *p* = .066, η^2^ = .12, but was not statistically significant. The main effect of hydration was not significant, *F*(1, 44) = 0.82, *p* = .37, η^2^ = .02, and there was no evidence of an interaction. Across hormonal groups, mean renin expression did not differ between EUHY and DEHY animals (see Figure 7E).

#### Uterine Weight

A two-way ANOVA examined the effects of hormonal group and hydration status on uterine weight. There was a significant main effect of hormonal group, *F*(2, 42) = 43.83, *p* < .001, η^2^ = .67, but no main effect of hydration, *F*(1, 42) = 0.01, *p* = .91, η^2^ < .01, and no interaction, *F*(2, 42) = 0.77, *p* = .47, η^2^ = .02. Post hoc Tukey's tests revealed that controls had greater uterine weight compared with OVX + Estradiol (*p* < .001) and OVX + Placebo (*p* < .001) animals (see Figure 7F). Additionally, OVX + Estradiol animals had higher uterine weight than did OVX + Placebo animals (*p* = .014; see Figure 7F).

## Discussion

This study examined how circulating estrogen and mild systemic DEHY influence USV profile in adult female rats using a food-reward elicitation paradigm. By combining ovariectomy, estradiol replacement, and hydration manipulations, we evaluated whether previously reported estrogen-related effects generalize to a food-reward USV paradigm and provide novel evidence that hormonal and hydration states exert selective and interactive effects on USV acoustic features. These effects were not uniform across measures, highlighting the complexity of endocrine and physiological regulation of vocalization.

Relative to controls, OVX animals produced shorter duration and reduced-power USVs. These effects were statistically significant but of small effect size, suggesting modest physiological changes. USV number and frequency range were largely preserved. A trend toward fewer USVs was observed in OVX animals, which is consistent with prior reports of diminished vocal output following ovariectomy (Garcia et al., 2017; Lenell & Johnson, 2021). Our findings of small changes to duration and power but not to USV numbers and frequencies extend previous reports that OVX alone does not consistently alter USV acoustic features when compared across the estrous cycle (Lenell et al., 2021; Lenell & Johnson, 2021). Temporal and amplitude-based parameters may be more sensitive to reduced circulating estrogen compared with spectral or quantity-based USV measures. From a laryngeal physiology perspective, USV duration and power depend primarily on sustained respiratory drive, precise regulation of glottal constriction, and neuromuscular control of laryngeal aperture rather than vocal fold vibration (Riede, 2013). Estrogen has well-documented effects on skeletal muscle metabolism, neuromuscular transmission, and epithelial integrity, which may preferentially influence the efficiency and endurance of respiratory–laryngeal coordination required to maintain stable airflow and glottal narrowing over time (Honda et al., 2016; Joshi et al., 2025; Sipilä et al., 2015; van der Giessen et al., 2019). Reduced circulating estrogen may therefore compromise the ability to sustain consistent subglottal pressure and aerodynamic sound generation, resulting in shorter duration and lower power USVs in OVX animals without necessarily altering USV initiation or frequency. In contrast, frequency-related measures may be more resilient, as USV frequency is largely determined by airflow velocity and glottal geometry and may be preserved through compensatory neural control of laryngeal constriction. However, it should be noted that small effect sizes were found in these measures, which may indicate limited functional relevance. Nonetheless, these subtle changes in vocal motor control could accumulate over repeated vocalization or under physiologically challenging conditions over long periods of time, leading to compromised voicing. These conditions and ensuing sequelae await further investigation.

Estradiol replacement successfully modulated systemic physiology, as confirmed by body and uterine weight measures (Gogos et al., 2018; Li et al., 2014; Smith et al., 2009; Zhang et al., 2020), but it did not fully restore a control-like USV profile. Instead, OVX + Estradiol animals emitted fewer USVs with shorter duration, reduced power, and narrower frequency range compared with controls. While these suppressive effects may appear counterintuitive, they align with prior work reporting context-dependent reductions in USV production under estradiol treatment (Garcia et al., 2017). In contrast, estradiol plus progesterone replacement has shown more restorative effects on USV features (Gerson et al., 2019; Matochik et al., 1992; McGinnis & Vakulenko, 2003), suggesting that estrogen alone may be insufficient to normalize laryngeal function. The nonrestorative and sometimes suppressive effects of estradiol highlight the importance of hormonal balance and potential synergistic roles of progesterone and other sex hormones. Together, these findings reinforce that hormonal effects on USVs are context dependent and underscore the importance of validating endocrine effects within specific behavioral paradigms before testing physiological interactions.

Hydration status further shaped USV outcomes, with DEHY reducing frequency range across hormonal groups and interacting with hormonal state to alter USV number and duration. These findings are consistent with prior work demonstrating that even mild systemic DEHY can impair laryngeal biology (Duan et al., 2021; Oleson et al., 2020) and extend this work by linking systemic hydration changes to USV acoustic consequences. However, DEHY may also influence behavior independently of laryngeal physiology. Animals experiencing mild DEHY may alter motivation, arousal, or energy allocation, potentially reducing vocal effort or changing vocalization strategies independent of direct effects on the larynx (Schwartz et al., 2026). Thus, DEHY-related changes in USVs may reflect a combination of peripheral laryngeal alterations and centrally mediated behavioral responses to physiological stress. Interestingly, controls reduced USV numbers comparing prehydration and posthydration challenge, whereas OVX groups often showed increases. These divergent patterns potentially suggest that estrogen status influences laryngeal sensitivity to systemic hydration. In intact controls, reduced USV production following hydration manipulation may reflect some adaptive energy conservation or altered reward valuation once physiological state is perturbed, even if hydration is restored. Conversely, OVX animals may exhibit heightened or dysregulated vocal responses due to altered motivational neural circuitry or reduced inhibitory control of vocal output (Curtis, 2009; Stachenfeld, 2008; Stachenfeld et al., 2001). Similarly, the shortening of complex USVs in euhydrated controls and lengthening in euhydrated OVX + Placebo animals may reflect differences in how hormonal state modulates the balance between vocal motor control and motivational drive. Complex USVs require greater respiratory–laryngeal coordination and sustained motor output (Riede, 2013). Intact animals may reduce this investment under changing physiological conditions, whereas hormone-manipulated animals may exhibit compensatory or less finely regulated vocal patterns. One possibility for this difference is that estrogen alters central reward-motivational pathways (Becker & Koob, 2016; Yoest et al., 2018), thereby influencing how animals prioritize food-seeking and related anticipatory vocalizations under physiological challenges (Garcia et al., 2017). Together, these findings suggest that estrogen status shapes both peripheral vocal system resilience and higher order control of vocal behavior. Future work incorporating direct laryngeal physiology measures and neural assessments could clarify the relative contributions of peripheral versus central mechanisms.

### Limitations and Future Directions

There are a few limitations that warrant consideration. First, many USV differences were of small effect size, which raises questions about their functional significance. As mentioned above, albeit small, these differences could suggest that an underlying emerging vulnerability of laryngeal function, which accrued over time, could manifest as more identifiable changes. Second, renin gene expression trended toward an increase but did not reach statistical significance. This finding highlights individual variability in physiological responses to systemic challenges in mammals. Importantly, renin transcription may remain unchanged during mild DEHY, even when RAAS activation occurs via rapid secretion of stored renin. Therefore, renin-based measures should be interpreted in conjunction with other physiological markers when confirming systemic DEHY. In the present study, imposed water restriction with a reduction in body weight served as the primary indicator confirming hydration manipulation. Third, USVs were elicited in a single anticipatory reward context. Estrogen and DEHY effects may vary depending on the type of environment (mating, social, stress, or isolation paradigms) in which USVs are elicited (Lenell et al., 2021). Fourth, although the 4- to 5-day estrous cycle was captured across recording periods, some control animals may have exhibited irregular cycles, which could contribute to variability in USV outcomes. Finally, only estradiol replacement was tested, limiting conclusions about the role of progesterone or combined hormone therapies. Future studies should investigate combined hormone replacement and longitudinal models of menopause to better capture clinically relevant hormonal transitions. Pairing USV analyses with direct laryngeal and neural measures will help disentangle peripheral from central contributions and clarify the conditions under which estradiol exerts restorative versus suppressive effects on laryngeal function during systemic challenges.

### Conclusions

This work provides new insight into how estrogen and hydration states interact to influence laryngeal function. Given the clinical relevance of reduced circulating estrogen and DEHY to vocal changes in aging women and clinical populations (e.g., menopause, hormone therapy, dysphonia), these findings establish an animal model for probing mechanisms underlying hormone and hydration interactions in the larynx. USVs offer a sensitive outcome for detecting even subtle vocal changes, offering translational insight into voice-related changes associated with menopause, hormone therapy, and hydration.

## Data Availability Statement

The data sets generated and/or analyzed during the current study are available from the corresponding author on reasonable request.
